# Comparison of Vibratome and Compresstome sectioning of fresh primate lymphoid and genital tissues for *in situ* MHC-tetramer and immunofluorescence staining

**DOI:** 10.1186/s12575-014-0012-4

**Published:** 2015-01-07

**Authors:** Hadia M Abdelaal, Hyeon O Kim, Reece Wagstaff, Ryoko Sawahata, Peter J Southern, Pamela J Skinner

**Affiliations:** Department of Veterinary and Biomedical Sciences, college of veterinary medicine, University of Minnesota, St. Paul, 1971 Commonwealth Avenue, Minnesota, MN 55108 USA; Departments of Microbiology, MMC 196, University of Minnesota, 420 Delaware Street SE, Minneapolis, MN 55455 USA; Departments of Microbiology and Immunology, Faculty of Pharmacy, Zagazig University, Zagazig, 44519 Egypt

**Keywords:** Compresstome, Vibratome, Unfixed fresh tissue sectioning, Vagina, Cervix, Uterus, Spleen, Lymph node, Immunohistochemistry, *in situ* tetramer staining

## Abstract

**Background:**

For decades, the Vibratome served as a standard laboratory resource for sectioning fresh and fixed tissues. In skilled hands, high quality and consistent fresh unfixed tissue sections can be produced using a Vibratome but the sectioning procedure is extremely time consuming. In this study, we conducted a systematic comparison between the Vibratome and a new approach to section fresh unfixed tissues using a Compresstome. We used a Vibratome and a Compresstome to cut fresh unfixed lymphoid and genital non-human primate tissues then used *in situ* tetramer staining to label virus-specific CD8 T cells and immunofluorescent counter-staining to label B and T cells. We compared the Vibratome and Compresstome in five different sectioning parameters: speed of cutting, chilling capability, specimen stabilization, size of section, and section/staining quality.

**Results:**

Overall, the Compresstome and Vibratome both produced high quality sections from unfixed spleen, lymph node, vagina, cervix, and uterus, and subsequent immunofluorescent staining was equivalent. The Compresstome however, offered distinct advantages; producing sections approximately 5 times faster than the Vibratome, cutting tissue sections more easily, and allowing production of larger sections.

**Conclusions:**

A Compresstome can be used to generate fresh unfixed primate lymph node, spleen, vagina, cervix and uterus sections, and is superior to a Vibratome in cutting these fresh tissues.

## Background

The capability of detecting cellular and microbial antigens in tissue sections has played an important role in understanding the cellular and molecular events surrounding microbial infections and innate and adaptive host immune responses. We have relied extensively on Vibratome sections of fresh unfixed rhesus macaque and human tissues for *in situ* MHC-tetramer staining of SIV-specific and HIV-1-specific CD8 T cells in lymphoid and genital tissues [1-5]. We also used a Vibratome to cut fresh tissues in a technique we developed for determining the quantitative and locative relationships between virus-specific CD8+ T cells (Effector cells) and infected cells (Targets). For this technique, *in situ* tetramer staining was combined with *in situ* hybridization to detect virus-specific CD8+ T and viral-RNA+ cells respectively [6,7].

In addition to genital [8] and lymphoid tissue [9], the Vibratome has been used to cut fresh tissue sections from brain [10,11], liver [12], kidney [13,14] larynx, thyroid and skeletal muscle tissues [15]. Despite these technical achievements, the standard Vibratome sectioning protocol retains two important disadvantages: the actual sectioning procedure is very time consuming and some tissues are particularly difficult to cut. For example, lymph node and spleen capsules are difficult to cut because the moving razor blade sometimes slides across the capsule connective tissue, rather than cutting through it, and this can lead to the tissue being pulled from the mount. Vagina and cervix tissues are difficult to cut because sometimes the blade catches on the tissue and pulls it out of the mount.

Tissue sectioning with the Vibratome is achieved by using a vibrating razor blade. The vibration amplitude, speed of blade movement, and blade angle are adjustable. The Vibratome can section either fresh or fixed tissue pieces with good preservation of ultra-structural tissue characteristics. Fresh Vibratome tissue sections are typically 200–500 microns thick [16,17] and fixed Vibratome tissue sections are typically 20 to 50 um thick [18,19]. The Compresstome™, VF-300 (Precisionary Instruments Inc., San Jose, California) is a new addition to the microtome family and can be used to cut fresh or fixed tissue sections. Viviani et al., used the Compresstome to cut fresh rat brain tissue [20]. The Compresstome was also used effectively to cut fresh rat liver [21] and lung tissue [22].

For sectioning tissues with the Compresstome, unfixed fresh tissue pieces are placed in a tube and embedded in low-melting temperature agarose and chilled immediately before sectioning. The specimen tube and the agarose both provide structural support to stabilize the tissue during sectioning. Furthermore, the specimen tube has an outlet of smaller diameter that makes a compression lip. So the tissue blocks are compressed when they are moved forward. These features of the Compresstome design improve both the quality of the sections and the efficiency of cutting.

In this study, we determined whether a Compresstome VF-300, (Precisionary Instruments Inc., San Jose, California) could be used to cut fresh unfixed genital and lymphoid tissues from rhesus macaques, and we compared the feasibility and efficiency of sectioning fresh unfixed genital and lymphoid tissues from rhesus macaques with the Vibratome 3000 (Technical Products International, St. Louis, MO) and the Compresstome VF-300. We cut 200 um-thick sections of fresh lymph node, spleen, vagina, cervix, and uterus from SIV-infected rhesus macaques and stained the tissue sections with MHC tetramer and immunofluorescent staining.

## Results and discussion

**Figure 1:**
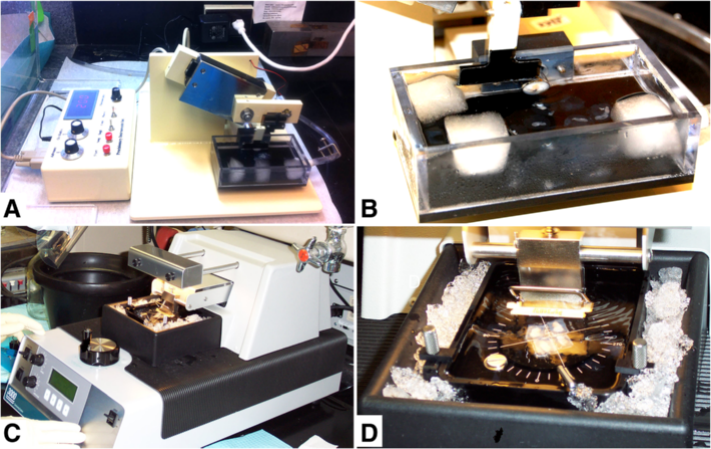
**Fresh tissue sectioning using a Compresstome and a Vibratome.** Cutting and chilling of fresh tissue with a Compresstome **(A-B)** and a Vibratome **(C-D)**.

Over the past decade, we have used Vibratomes extensively to section fresh unfixed lymphoid and genital tissues (Figure 1) from over 200 rhesus macaques and performed *in situ* tetramer staining combined with immunohistochemistry to develop a temporal and spatial understanding of the progression of SIV infection and the distribution of induced SIV-specific CD8 T cells [1,3-7]. More recently, we have used the Compresstome to section fresh unfixed tissues (Figure 1) from nearly 50 rhesus macaques for the combined *in situ* tetramer and immunohistochemistry staining (unpublished data). Here we compare the feasibility and efficiency of sectioning fresh genital and lymphoid tissues from rhesus macaques with the Vibratome 3000 and the Compresstome VF-300.

The time required for sample preparation immediately prior to sectioning was approximately similar for the two methods. Both instruments allowed some element of temperature control during sectioning of fresh tissues (Figure 1 and Table 1). We have found that chilling fresh tissue prior to sectioning is essential for obtaining high quality sections. Appropriate temperature control is not only central to successful sectioning of fresh unfixed tissue samples, but is also a safeguard for retention of cell viability and halting the metabolic process in the tissue being sectioned.

**Table 1:** **Comparison between the Compresstome VF-300 and the Vibratome 3000**

	**Compresstome VF-300**	**Vibratome 3000**
Presence of light source	No	Yes
Construction	Flimsy	Solid
Cutting speed	Fast ~5 sections/minute	Slow ~0.8 sections/minute
Number of blocks that can be cut at the same time	One	Four
Chilling capability	Frozen buffer cubes added to the buffer tank	Dry ice or ice placed in the outer chamber
Sectioning difficulties due to blade dislodging embedded tissue	Rare	Frequent
Maximum section size	~0.64 cm^2^	~0.25 cm^2^

In sectioning with the Compresstome, we found that we could generate fresh tissue sections much more quickly compared to the Vibratome. Cutting tissues with the Vibratome required that the blade move forward at an extremely slow, almost undetectable speed. In fact, in about half of the six Vibratomes that we have purchased over the years, we needed to adjust the machines so that the slowest forward motion speed was substantially slower than the factory set slowest speed. In contrast, the Compresstome, by virtue of offering more support to the tissue block while cutting, allowed sections to be produced more quickly, with a much faster cutting speed. We cut approximately 5 sections per minute using a Compresstome. Whereas, when cutting 4 tissue blocks simultaneously using the Vibratome, we obtained only about 0.8 sections per minute (Table 1). Thus, we generated fresh lymphoid and genital tissue sections over 5X faster with the Compresstome than the Vibratome. This increased throughput with the Compresstome was highly advantageous in processing multiple tissue samples.

One problem we experienced while sectioning with the Vibratome was that the moving blade would frequently catch on a fibrous region of tissue such as the lymph node capsule, and dislodge the entire tissue piece. Displacement of the embedded tissue was particularly problematic when cutting fresh vagina and cervix tissues with the Vibratome. We rarely experienced this problem using the Compresstome. We also found that the structural stability of the embedded tissue offered by the Compresstome tube allowed for up to about 60% larger sized sections (0.64 cm^2^ Vs. 0.25 cm^2^) compared to Vibratome sections (Table 1). Thus, the Compresstome was superior to the Vibratome in the ease of generating lymphoid and genital sections and allowing the generation of larger sections.

**Figure 2:**
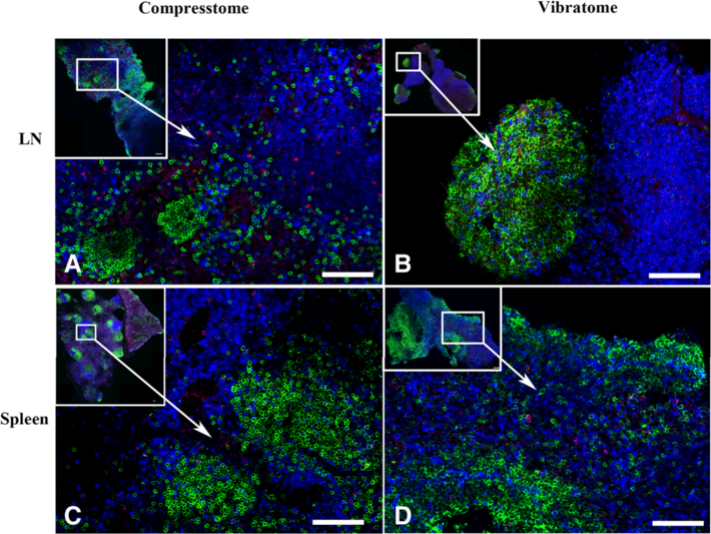
**Comparison of fresh lymphoid tissue sections cut with a Compresstome or Vibratome.** Fresh unfixed lymph node **(A-B)** and spleen **(C-D)** tissue sections were generated with either a Compresstome (left panels) or Vibratome (right panels) and processed for *in situ* tetramer staining combined with immunohistochemistry. Tissue sections were stained with *Mamu-A1*/Gag (CM9) MHC-tetramers to label SIV-specific CD8 T cells (red to pinkish color), anti-CD20 antibodies to detect B cells (green), and anti-CD3 antibodies to detect T cells (Blue). Confocal mages were collected using a 20 X objective and 3 um z-steps. The upper left panel of each image shows a montage of several projected confocal z-series fields. Scale bars = 100 microns. We used the curves tool in Photoshop to increase the contrast of each image, and each image was adjusted similarly.

**Figure 3:**
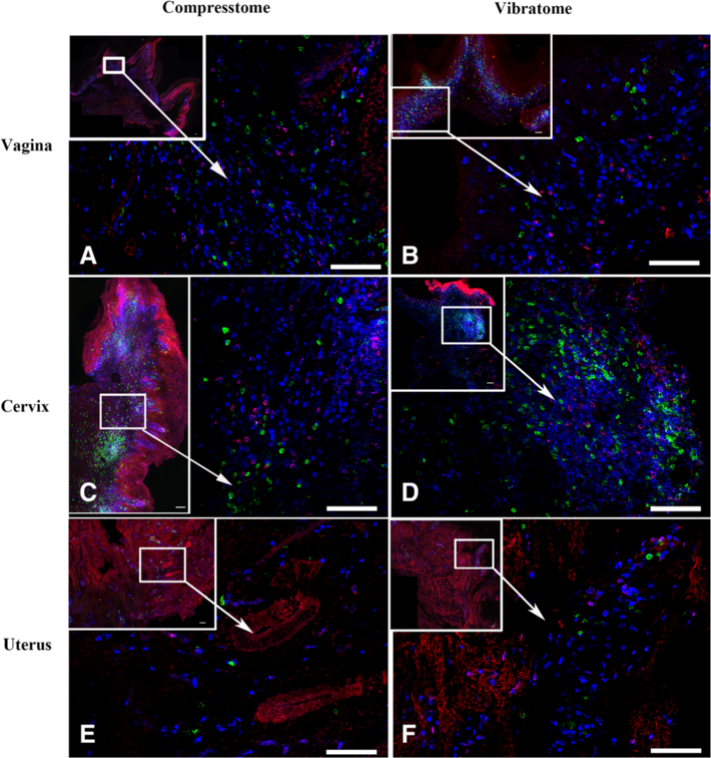
**Comparison of fresh genital tissue sections cut with a Compresstome or Vibratome.** Fresh vagina **(A-B)**, cervix **(C-D)** and uterus **(E-F)** tissue sections generated with either a Compresstome (left panels) or Vibratome (right panels) and processed for *in situ* tetramer staining combined with immunohistochemistry. Tissue sections were stained with *Mamu-A1*/Gag (CM9) MHC-tetramers to label SIV-specific CD8 T cells (red to pinkish color), anti-CD20 antibodies to detect B cells (green), and anti-CD3 antibodies to detect T cells (Blue). Confocal mages were collected using a 20 X objective and 3 um z-steps. The upper left panel of each image shows montage of several projected confocal z-series fields. Scale bars = 100 microns. We used the curves tool in Photoshop to increase contrast of each image, and each image was adjusted similarly.

The quality of the resultant confocal microscope images obtained from the stained tissue sections was similar for the two sectioning procedures for both lymphoid tissues and genital tissues. Figure 2 shows spleen and lymph node, and Figure 3 shows vagina, cervix and uterus tissue sections generated with either a Compresstome (left panels) or Vibratome (right panels) stained with *Mamu-A1*/Gag (CM9) MHC-tetramers to label SIV-specific CD8 T cells, and anti- CD20 and anti-CD3 antibodies to detect B cells and T cells, respectively.

While we found the Compresstome superior to the Vibratome with regard to generating fresh tissue sections more quickly and easily, we found that the overall construction of the Compresstome to be inferior to the Vibratome. First, the Compresstome construction is relatively flimsy and it has a much less sleek appearance than the Vibratome (Figure 1). In addition, we encountered a problem with the Compresstome that stemmed from not being able to reverse the forward motion of the blade. This problem occurred when we inadvertently ran the blade into the plastic tube that holds the tissue. We were not able to reverse the forward motion of the blade and the blade became jammed in the plastic. Thus, for these reasons, we found the overall construction of the Compresstome to be inferior to the Vibratome.

## Conclusions

A Compresstome can be used to generate fresh unfixed primate lymphoid and genital tissue sections of high quality. In addition, the use of a Compresstome for sectioning fresh genital and lymphoid tissues offers advantages over the Vibratome including 1) generating fresh tissue sections approximately five times faster, 2) ability to cut slightly larger sized tissues sections, and 3) cutting difficult tissues such as genital tract tissues with ease. Thus, we recommend using a Compresstome for cutting fresh primate lymph node, spleen, vagina, uterus, and cervix tissues.

## Materials and methods

### Animals and collection of tissues

Adult female Indian rhesus macaques (Macaca mulatta) expressing Mamu- A1*001:01 or Mamu-B*008:01 MHC class I alleles [23] were housed at BIOQUAL, Inc. (Rockville, MD), the California National Primate Research Center, the Wisconsin National Primate Research Center, or the New England Primate Center (NEPRC) and maintained according to the regulations of the American Association of Accreditation of Laboratory Animal Care standards following Institutional Animal Care and Use Committee approved procedures. Animals were infected intra- rectally or intra-vaginally with SIVmac251 or SIVmac239 and were euthanized as we have described [1,3-5,24]). Freshly dissected genital tissues including, vagina, cervix, uterus, ovary and lymphoid tissues including axillary, mesenteric, inguinal lymph nodes and spleen, were collected from animals after euthanasia. Fresh tissues were placed into tubes containing chilled RPMI with 100 mg/ml of heparin (heparin acts as an RNase inhibitor) and were shipped on ice overnight to the University of Minnesota.

### Sectioning of fresh unfixed tissue using a Vibratome

Fresh tissues were sectioned as previously described [9,25] using a Vibratome 3000 (Technical Products International, St. Louis, MO). Fresh tissues were cut with a surgical scalpel into small, ~0.5-cm-wide × 0.5-cm-tall pieces. Tissue pieces were put in a weigh boat and covered melted 4% PBS-buffered low-melt agarose at 40C and allowed to solidify on ice. The Vibratome bath was then filled with ice-cold sterile phosphate buffer saline- containing 100mg/ml heparin (PBS-H) (we added heparin as an inhibitor of RNase for subsequent procedures not described here). A half of a double edge razor blade (Persona Super Blade, American Safety Razor Company, Verona, Va) was inserted into the blade holder and blade angle was adjusted to 27 degrees. The Vibratome bath was maintained at 0-2C using ice or dry ice surrounding the Vibratome bath chamber as shown in Figure 1. The resulting agarose block containing the tissue piece was then glued to a metal mounting block using Loctite® 404® Quick SetTM Instant Adhesive (Henkel) and sectioned while submerged in the buffer bath. Individual 200um thick sections were collected with a fine brush and transferred to 24 multi-well plates for staining.

### Sectioning of fresh unfixed tissue using a Compresstome

Fresh tissues were cut with a surgical scalpel into ~0.8-cm-wide × 0.8-cm-tall pieces and were glued onto the cutting plunger of a specimen syringe using Loctite® 404® Quick SetTM Instant Adhesive. Tissues then were pulled down into the plunger and covered with melted ~40C PBS-H-buffered low melt agarose, and the plunger with agarose and tissue was placed into a chilled block where the agarose solidified. A half of a double edge razor blade was glued with Loctite Instant Adhesive to the blade holder, and attached to the machine. The plunger containing the embedded tissue was then inserted into the cutting chamber of the Compresstome. The buffer tank was filled with solution of chilled PBS-H followed by addition of 3 frozen cubes of PBS-H to maintain 0C (Figure 1). Then the razor blade edge was closely aligned to the outlet of the specimen syringe, the thickness of each section was set to 200um, and then the instrument was turned on to automatically cut slices. Tissue sections were harvested from the buffer chamber using a brush.

### In situ tetramer staining combined with immunohistochemistry

*In situ* tetramer staining combined with immunohistochemistry was done as previously described [5,9,25]. Biotinylated molecules of Mamu-A1*001:01 loaded with SIV Gag (181– 189) CTPYDINQM (CM9) peptides (will be referred to as *Mamu-A1*/Gag hereafter) and of *Mamu-A1* loaded with an irrelevant peptide FLPSDYFPSV (FLP) from the hepatitis-B viral protein (served as a negative control) were obtained from the NIH Tetramer Core Facility. MHC tetramers were prepared as previously described [9], by mixing MHC monomers with 6 aliquots of FITC-labeled Extra Avidin (Sigma, St. Louis, MO) over 8 hours to a final molar ratio of 1:4.5. For *in situ* tetramer and antibody staining, fresh tissue sections were incubated at 4C overnight with MHC class I tetramers (0.5 ug/ml) in 1 ml of PBS containing 100mg/ml heparin (PBS-H) with 2% normal goat serum (NGS). Sections were then washed with chilled PBS-H followed by fixation with freshly prepared 4% paraformaldehyde for 2 hours at RT and washed with chilled PBS-H. For antigen retrieval, sections were boiled 3 times in urea and permeabilized and blocked with PBS-H containing 0.3% triton x-100 0.3% and 2% NGS for 1 hour prior to secondary incubation. A secondary incubation was done overnight at 4°C with rabbit anti-FITC antibodies (BioDesign, New York, NY) diluted 1:10,000, rat-anti-human CD3 antibodies (AbD Serotec clone CD3-12, Raleigh, NC) diluted 1:200, and mouse anti-CD20 antibody clone L26, diluted 1:200 (Novocastra, Leica Microsystems Inc., Buffalo Grobe, IL) in PBS-H containing 2% NGS on rocking platform. Tissue sections were then washed with chilled PBS-H and incubated with Cy3-conjugated goat anti-rabbit antibodies diluted 1:5000 (Jackson ImmunoResearch, West Grove, PA), Cy5-conjugated anti-rat antibody (Jackson ImmunoResearch, West Grove, PA), diluted 1:2000, and Alexa 488-conjugated goat anti-mouse antibody (Molecular Probes, Eugene, OR) diluted 1:2000 in blocking solution for 1–3 days. After the final antibody incubation, tissue sections were washed with PBS-H and post-fixed for 1 hour with 4% paraformaldehyde. Sections were then washed with PBS-H and mounted on slides with warm glycerol gelatin (Sigma, St. Louis, MO) containing 4-mg/ml n-propylgallate (as a fluorophore preservative).

### Confocal imaging

Confocal images were obtained using an Olympus FluoView 1000 confocal microscope (Olympus Corporation, Tokyo Japan), a 20x objective and z-steps of 3 um. Images were collected from approximately 6 um below the surface of the tissue section to the limit of penetration of the antibody counter staining, usually about 36–42 um into the interior of the tissue sections. We used Olympus FluoView Viewer software to create montage images of multiple 800x800 pixels 200X Z-scans.
